# Deep Kernel and Deep Learning for Genome-Based Prediction of Single Traits in Multienvironment Breeding Trials

**DOI:** 10.3389/fgene.2019.01168

**Published:** 2019-12-09

**Authors:** José Crossa, Johannes W.R. Martini, Daniel Gianola, Paulino Pérez-Rodríguez, Diego Jarquin, Philomin Juliana, Osval Montesinos-López, Jaime Cuevas

**Affiliations:** ^1^ Biometrics and Statistics Unit, Genetic Resources Program, and Global Wheat Program, International Maize and Wheat Improvement Center (CIMMYT), Texcoco, Mexico; ^2^ Department of Animal Sciences, University of Wisconsin-Madison, Madison, WI, United States; ^3^ Programa de Postgrado de Socioeconomia, Estadistica e Informatica, Colegio de Postgraduados, Texcoco, Mexico; ^4^ Department of Agronomy and Horticulture, University of Nebraska-Lincoln, Lincoln, NE, United States; ^5^ Facultad de Telemática, Universidad de Colima, Colima, Mexico; ^6^ Departamento de Ciencias, Universidad de Quintana Roo, Chetumal, Mexico

**Keywords:** deep learning, deep kernel, genomic selection, kernel methods, artificial neural networks, genomic × environment interaction

## Abstract

Deep learning (DL) is a promising method for genomic-enabled prediction. However, the implementation of DL is difficult because many hyperparameters (number of hidden layers, number of neurons, learning rate, number of epochs, batch size, etc.) need to be tuned. For this reason, deep kernel methods, which only require defining the number of layers, may be an attractive alternative. Deep kernel methods emulate DL models with a large number of neurons, but are defined by relatively easily computed covariance matrices. In this research, we compared the genome-based prediction of DL to a deep kernel (arc-cosine kernel, AK), to the commonly used non-additive Gaussian kernel (GK), as well as to the conventional additive genomic best linear unbiased predictor (GBLUP/GB). We used two real wheat data sets for benchmarking these methods. On average, AK and GK outperformed DL and GB. The gain in terms of prediction performance of AK and GK over DL and GB was not large, but AK and GK have the advantage that only one parameter, the number of layers (AK) or the bandwidth parameter (GK), has to be tuned in each method. Furthermore, although AK and GK had similar performance, deep kernel AK is easier to implement than GK, since the parameter “number of layers” is more easily determined than the bandwidth parameter of GK. Comparing AK and DL for the data set of year 2015–2016, the difference in performance of the two methods was bigger, with AK predicting much better than DL. On this data, the optimization of the hyperparameters for DL was difficult and the finally used parameters may have been suboptimal. Our results suggest that AK is a good alternative to DL with the advantage that practically no tuning process is required.

## Introduction

Using dense molecular markers, Meuwissen et al. (2001) were the first to propose genome-enabled prediction for implementing genomic-assisted breeding. Subsequently, an enormous number of research articles published in animal and plant breeding journals explored and studied genomic selection (GS) and genome-based prediction (GP) outcomes in a large variety of animal and plant species for different traits and measured in different environments (Crossa et al., 2017). GS combines molecular and phenotypic data in a training population to predict genomic breeding values (or genetic values) of individuals that have been genotyped but not phenotyped. The predictions can be used in a breeding program to reduce cycle length or to increase the selection precision, thus enhancing the response to selection.

GS and prediction approaches have focused on two different cases. One is predicting additive effects in early generations of a breeding program to achieve rapid selection with a short interval cycle (Beyene et al., 2015; Zhang et al., 2017). Here, breeders focus on GP of breeding values (additive values) of an infinitesimal model that assumes a linear function of allelic effects for an infinite number of loci; therefore, additive linear models that summarize the effects of the markers are sufficient. The most commonly used additive method is genomic best linear unbiased predictor (GBLUP/GB) (Van Raden, 2007). The other case considers the complete genetic values of individuals including both additive and nonadditive (dominance and epistasis) effects, thereby estimating the genetic performance of the cultivars (Crossa et al., 2017).

As pointed out by Harfouche et al. (2019), despite the fact that GS programs have provided extensive amounts of new data in crops, legumes, and tree species, the lack of predictive accuracy for many complex traits is underpinned by the complexity of modeling all of the important factors inherent to targets such as grain yield. Harfouche et al. (2019) mentioned that linking phenotypes with genotypes using high-throughput phenomics and genomics will continue to be the main challenge for plant breeding in the next decades.

The complexity of applying GS and GP in breeding is influenced by various factors acting at different levels. An important difficulty arises when predicting unobserved individuals in specific environments (site-year combinations) by incorporating genotype (genomic) × environment (G×E) interaction into statistical models. An additional layer of complexity is the G×E interactions for multitraits. Here statistical-genetic models exploit multitrait, multienvironment variance-covariance structures and correlations between traits and environments simultaneously. Understanding the complexity of traits requires a theoretical framework that accounts for often cryptic interactions.

Some of the statistical complexities can be addressed by using semiparametric genomic regression methods to account for nonadditive variation (Gianola et al., 2006; Gianola et al., 2011; Morota and Gianola, 2014; Morota et al., 2014). These methods have been used to predict complex traits in wheat with promising practical results (González-Camacho et al., 2012; Pérez-Rodríguez et al., 2012). Semiparametric models often use kernel methods (a kernel utilizes functions that represent the inner product of many basic functions) for addressing complex gene actions (e.g., gene×gene epistatic interactions), thus capturing nonlinear relations between phenotype and genotype. Kernel-based methods for genomic regression have been used extensively in animal and plant breeding due to their capacity to produce reasonably accurate predictions (Gianola et al., 2014).

A commonly used kernel is the Gaussian kernel (GK) defined as $\exp(-hd_{ii'}^{2}/q)$, where *h* is a bandwidth parameter which controls the rate of decay of the covariance between genotypes, and *q* is the median of the square of the Euclidean distance, $d_{ii'}^{2}={\sum_{k}\left(x_{ik}-x_{i'k}\right)}^{2}$ which is a measure of the genetic distance between individuals (*i,i’*) based on molecular markers. The parameter *q* could also be included in the bandwidth parameter *h*, but standardizing the Euclidean distances by *q* makes it easier to apply a standardized grid search when looking for the optimal *h*. The GK appears as a reproducing kernel in the semiparametric reproducing kernel Hilbert spaces (RKHS) (Gianola and van Kaam, 2008; González-Camacho et al., 2012). Pérez-Elizalde et al. (2015) proposed an empirical Bayes method for estimating the bandwidth parameter *h*. An alternative approach to using a kernel with specific bandwidth parameters is the multikernel fitting proposed by de los Campos et al. (2010). Cuevas et al. (2016; 2017; 2018) and Souza et al. (2017) showed that using the GK within the multienvironment genomic G×E model of Jarquín et al. (2014) led to higher prediction accuracy than the same method with the linear kernel GB. Parametric alternatives for modeling epistasis have also been broadly discussed in literature (Jiang and Reif, 2015; Martini et al., 2016).

Deep learning (DL) methods are very flexible and have the potential to adapt to complex potentially cryptic data structures. In general, DL architectures are composed of three types of layers: (1) an input layer corresponding to the input information (predictors, that is, markers); (2) hidden layers, that is, the number of internal transformations performed on the original input information, which can be at least one but also a larger number; however, the number of neurons in each hidden layer needs to be tuned or specified; and (3) the output layer that produces the final predictions of the response variables we are interested in. Montesinos-López et al. (2018a; 2018b; 2019a; 2019b) recently performed extensive studies using DL methods for assessing GP for different types of traits (continuous, ordinal, and binary) accounting (or not) for G×E and comparing their prediction accuracies with those obtained by GB for single environments and multiple environments (with G×E). The authors used data from extensive maize and wheat multitrait, multienvironment trials. DL produced similar or slightly better prediction accuracies than GBLUP when G×E was not considered, but it was less accurate when G×E was included in the model. The authors hypothesized that DL may already account for G×E, so that its inclusion in the model was not required. Overall, the current drawback of applying DL for GP is the lack of a formal method for defining hyperparameters (e.g., number of neurons, number of layers, batch size) and, therefore, the time required for parameter tuning. Moreover, there may be an increased tendency towards overfitting the training data, and when important data features such as G×E interaction are known, direct modeling may lead to better predictions than modeling the structures implicitly in DL.

Recently, Cuevas et al. (2019) introduced the positive-definite arc-cosine kernel (AK) function for genome-enabled prediction. The AK was initially proposed by Cho and Saul (2009) for exploring the option of DL in kernel machines. The nonlinear AK is defined by a covariance matrix that emulates a DL model with one hidden layer and a large number of neurons. Moreover, a recursive formula allows altering the covariance matrix stepwise, thus adding more hidden layers to the emulated deep neural network. The AK kernel method has been used in genomic single-environment models, as well as for genomic multienvironment models including genomic × environment interaction (G×E) (Cuevas et al., 2019). AK has the advantage over GK that it is computationally much simpler, since no bandwidth parameter is required, while performing similarly or slightly better than GK. The tuning parameter “number of layers” which is required for AK can be determined by a maximum marginal likelihood procedure (Cuevas et al., 2019).

Although AK has already been compared with GK (Cuevas et al., 2019), AK has not been formally compared with DL methods. Therefore, the main objective of this study was to compare the genome-based prediction accuracy of the GB, GK, AK, and DL methods using single-environment and multienvironment G×E models on two data sets from the CIMMYT Global Wheat Program. The data sets comprised two years (2015–2016 and 2016–2017) of Elite Yield Trial data, each consisting of 1052 and 1040 elite wheat lines, respectively. Lines of both Elite Yield Trials were evaluated in four environments using two irrigation levels [5 irrigations, 5IR, and 2 irrigations, 2IR] and two planting systems (flat, F, and bed, B) reflecting mega-environments defined by breeders in South Asia and Mexico.

## Material and Methods

### Genome-Based Prediction Models

The statistical methods used in this study have been described in several articles (Cuevas et al., 2016; Cuevas et al., 2017; Souza et al., 2017; Cuevas et al., 2018) for the single-environment model and the multienvironment G×E models using the GB and the GK. In addition, AK has recently been described in Cuevas et al. (2019). A brief description of the models (single-environment and G×E models) and methods (GB, GK, AK, and DL) is given below.

#### Single-Environment and Multiple-Environment G×E Models

For a single environment and only one kernel, the model can be expressed as:

(1) y=μ1+u+ε

where *µ* is the overall mean, **1** is the vector of ones, and ***y*** is the vector of observations of size *n*. Moreover, ***u*** is the vector of genomic effects $u\sim N(0,\sigma_{u}^{2}K)$, where $\sigma_{u}^{2}$ is the genomic variance estimated from the data, and matrix ***K*** is constructed as $K=Z_{g}GZ_{g}^{'}$, with matrix ***Z*** ***_g_*** a matrix of 0s and 1s with exactly one 1 in each row, and which relates the genotypes to the observations. The covariance matrix ***G*** models the genomic similarities between genotypes and varies between models: GB (***G***=***XX’***/*p*) (where ***X*** is the scaled marker matrix and *p* denotes the number of markers); GK ($G_{ii'}=\exp(hd_{ii'}^{2}/q)$ where $d_{ii'}^{2}=\sum_{k}{\left(x_{ik}-x_{i'k}\right)}^{2}$); and AK (see the description below). The random residuals are assumed independent with normal distribution $\varepsilon \sim N(0,\sigma_{\varepsilon }^{2}I)$, where $\sigma_{\varepsilon }^{2}$ is the error variance.

In the G×E multienvironment model of Jarquín et al. (2014), Lopez-Cruz et al. (2015), and Cuevas et al. (2016), Eq. (1) takes the form

(2) $$y=\mu 1+Z_{E}\beta_{E}+u_{1}+u_{2}+\varepsilon$$

where ***y***=[***y***_1_, ,***y*** *_nm_*]’ are the observations collected in each of the *m* sites (or environments) with *n* lines in each site. The fixed effects of the environment are modeled with the incidence matrix of environments ***Z*** *_E_*, where the parameters to be estimated are the intercept for each environment ***β*** *_E_* (other fixed effects can be incorporated into the model). In this model, $u_{1}\sim N\left(0,\sigma_{u_{1}}^{2}K_{1}\right)$ represents the genomic main effects, $\sigma_{u_{i}}^{2}$ is the genomic variance component estimated from the data, and $K_{1}=Z_{g}GZ_{g}^{'}$, where ***Z*** *_g_* relates the genotypes to the phenotypic observations. The random effect ***u***_2_ represents the interaction between the genomic effects and their interaction with environments and is modeled as $u_{2}\sim N\left(0,\sigma_{u_{2}}^{2}K_{2}\right)$, where $K_{2}=\left(Z_{g}GZ_{g}^{'}\right){}^{\circ}(Z_{E}Z_{E})$, where ° is the Hadamard product.

#### AK Method

DL architectures are generally difficult to tune. The tuning process involves, for instance, selecting the activation function, determining the number of hidden layers and the number of neurons in each hidden layer, and selecting the type of regularization. For this reason, Neal (1996) proposed a Bayesian method for deep artificial neural networks (ANN with more than one hidden layer), also called simple DL models, and, in conjunction with the results of Williams (1998) and Cho and Saul (2009), established the relationship between the AK and the deep neural networks with one hidden layer. These authors proposed a family of kernels that emulate DL models.

For AK, an important component is the angle between two vectors computed from marker genotypes ***x*** *_i_* ***x*** *_i_*, as

$$\;\;\theta_{i,i^{\prime }}=\cos^{-1}\left(\frac{x_{i}\cdot x_{i^{\prime }}}{\left||x_{i}||||x_{i^{\prime }}|\right|}\right)$$

where ˙ denotes the inner product and ||***x*** ***_i_***|| is the norm of line *i*. The following kernel is positive semidefinite and related to an ANN with a single hidden layer and the ramp activation function (Cho and Saul, 2009)

(3) $$AK^{1}(x_{i},x_{i'})=\frac{1}{\pi }||x_{i}||||x_{i'}||J(\theta_{i,i'})$$

where *π* is the pi constant and *J*(*θ* *_i,i’_*)=[sin(*θ* *_i,i’_*)+(π-*θ* *_i,i’_*)cos(*θ* *_i,’i_*)]. Equation (3) gives a symmetric positive semidefinite matrix (*AK* ^1^) preserving the norm of the entries such that AK(***x*** *_i_*, ***x*** *_i_*)=||***x*** *_i_*||^2^, and AK(***x*** *_i_*, - ***x*** *_i_*)=0 and models nonlinear relationships.

Note that the diagonal elements of the AK matrix are not identical and express heterogeneous variances of the genetic values *u*. This is different from the GK matrix, with a diagonal that expresses homogeneous variances. This property could represent a theoretical advantage of AK when modeling interrelationships between individuals.

In order to emulate the performance of an ANN with more than one hidden layer (*l*), Cho and Saul (2009) proposed a recursive relationship of repeating *l* times the interior product

(4) $$AK^{\left(l+1\right)}(x_{i},x_{i^{\prime }})=\frac{1}{\pi }{\left[AK^{\left(l\right)}(x_{i},x_{i})AK^{\left(l\right)}\left(x_{i^{\prime }},x_{i^{\prime }}\right)\right]}^{\frac{1}{2}}\;J(\theta_{i,i'}^{\left(l\right)})$$

where $\theta_{i,i'}^{\left(l\right)}=\cos^{-1}\{AK^{\left(l\right)}(x_{i},x_{i^{\prime }}){\left[AK^{\left(l\right)}(x_{i},x_{i})AK^{\left(l\right)}(x_{i^{\prime }},x_{i^{\prime }})\right]}^{-\frac{1}{2}}\}$. Thus, computing *AK* ^(^ *^l^* ^+1)^ at level (layer) *l*+1 is done from the previous layer *AK* ^(^ *^l^* ^).^ Computing a bandwidth is not necessary, and the only computational effort required is to compute the number of discrete layers. Cuevas et al. (2019) described a maximum marginal likelihood method used to select the number of hidden layers (*l*) for the AK kernel.

#### DL Neural Network

The DL for a single trait, including the multienvironment G×E situation employed in this study, follows the approach delineated by Montesinos-López et al. (2018a). In DL, the input to the model is a vector space that is subject to several complex geometric transformations that decompose into simple geometric transformations. The main objective of these geometric transformations is to map the input space to the target output space where the transformations are parameterized by the weight of the input at each neuron in each layer. A brief description of the process for tuning DL and for model selection is provided.

The implemented DL has a feedforward topology in which the information moves in only one direction (i.e., forward) from the input nodes (representing prediction variables), through the hidden nodes (located in hidden layers), and to the output nodes (representing target variables). There are no cycles or loops in this network. The three groups of nodes in this DL model are called layers. When the DL model has only one hidden layer, it reduces to a conventional artificial neural network. The lines connecting the input layer neurons, hidden layer neurons, and output layer neurons represent the network weights which need to be learned. From all input connections, the hidden neuron sums up the corresponding weight so the weighted summation is then transformed through an activation function to produce the output of each neuron. The activation functions play an important role in transforming the input and output of hidden layers so they come out in a more useful form (Chollet and Allaire, 2017).

We used the rectified linear unit (RELU) as the activation function for all neurons in the hidden and output layers because our response variables are continuous. In addition, we used a batch size of 56 for implementing the DL model with 1,000 epochs. One epoch means one pass (forward and backward) of the full training set through the neural network, and to complete an epoch, we required a certain number of iterations calculated as the size of the training set divided by 56 (batch size). We used the R statistical software (R Core Team, 2019) for implementing all the models, and the DL model was implemented in the keras library (Chollet and Allaire, 2017). In keras we used the root-mean-square propagation (RMSprop) method with its default values as an optimizer. Also, to avoid overfitting we used dropout regularization, which consists of temporarily removing a random subset (%) of neurons with their connections during training.

For selecting the number of hidden layers, the number of units (neurons) in each hidden layer and the % dropout that needs to be defined, we used a grid search method. In grid search, each hyperparameter of interest is discretized into a desired set of values to be studied, and models are trained and assessed for all combinations of the values across all hyperparameters (that is, a “grid”). The grid search looked for the optimal combination of these three hyperparameters; the values used in the grid were 1, 2, 3, and 4 hidden layers. With regard to the number of units, we tried 80, 160, 240, 320, and 400 units, while for the % dropout (% neurons removed from the DL network), we tried 0%, 5%, 10%, 20%, 25%, and 35%. To select the optimal combination of these three hyperparameters, we implemented a fivefold cross-validation. After obtaining the optimal combination of hyperparameters, the model was refitted using the complete training data.

### Random Cross-Validations for Assessing Model Prediction Accuracy

The cross-validation strategy used in this study was a fivefold random cross-validation where 20% of the wheat lines were predicted by 80% of the other lines. This is the random cross-validation CV2 (Burgueño et al., 2012) that mimics a prediction problem faced by breeders in incomplete field trials where lines are evaluated in some, but not all, target environments (usually called *sparse testing*, when not all breeding lines are included for testing in all the environments). In this case, 20% of the lines are not observed in some environments and thus predicted in those environments, but are observed in other environments. When the main purpose of the model is prediction, a reasonable criterion of model quality is the mean squared error of prediction (MSEP) that measures the mean squared distance between the prediction value and the observed value.

Predictions were made for each environment for both the single-environment model (G) and the G×E multienvironment model, using GB, GK, and AK constructed with molecular markers. To make the models comparable in their prediction accuracy as well as their computing time, exactly the same random cross-validations were used for the four methods: GB, GK, AK, and DL.

### Experimental Data

We used data from CIMMYT’s Global Wheat Program (GWP) consisting of a set of elite wheat lines evaluated under differently managed environmental conditions at CIMMYT’s main wheat breeding experiment station in Cd. Obregon, Mexico. These environmental conditions simulated target areas of megaenvironments for the CIMMYT GWP. The wheat lines included in this study were later included in screening nurseries that were distributed worldwide.

#### Phenotypic Data

The phenotypic data consist of grain yield (ton/ha) records collected during two evaluation years (year 2015–2016 including 1,052 elite wheat lines, and year 2016–2017 including 1,040 elite wheat lines). All trials were established using an alpha-lattice design with three replicates per line and environment. Each environment was defined by a combination of a planting system (BED = bed planting; FLAT = planting on the flat) and an irrigation intensity (2IR = two irrigations giving moderate drought stress; 5IR = five irrigations representing an optimally irrigated crop). In the 2IR and 5IR regimes, irrigation was applied without measuring soil moisture, and each irrigation added 100 mm of water. Thus, for each of the years (2015–2016 and 2016–2017), four environments BED5IR, FLAT5IR, BED2IR, and FLAT2IR were established. The phenotype used in the analysis was the best linear unbiased estimate (BLUE) of grain yield obtained from a linear model applied to the alpha-lattice design of each year-environment combination. The data included in the present study represent two years of field trials under the same environmental conditions and using similar experimental designs. However, the wheat lines included in both data sets are different and the environmental conditions of the two years were relatively different during the growing season. We therefore decided not to consider a joint analysis of the two data sets.

#### Genotypic Data

Genotypes were derived using genotyping-by-sequencing technology (GBS; Poland et al., 2012). GBS markers with a minor allele frequency lower than 0.05 were removed. As is typical of GBS genotypes, all markers had a high uncalling rate. In our quality control pipeline, we applied thresholds for the incidence of missing values aimed at maintaining relatively large and similar numbers of markers per data set. We removed markers with more than 60% missing values; this left 15,744 GBS markers available for analysis. Finally, only lines with more than 2,000 called GBS markers were used in the data analysis; this left 515 and 505 wheat lines in years 2015–2016 and 2016–2017, respectively.

### Data Repository

The phenotypic and genotypic data for both data sets, year 2015–2016 and year 2016–2017, are available at the following link: http://hdl.handle.net/11529/10548273. Furthermore, basic codes for running the DL and AK kernel methods can be found in the **Appendix**.

## Results

### Phenotypic Data

**Figure 1:**
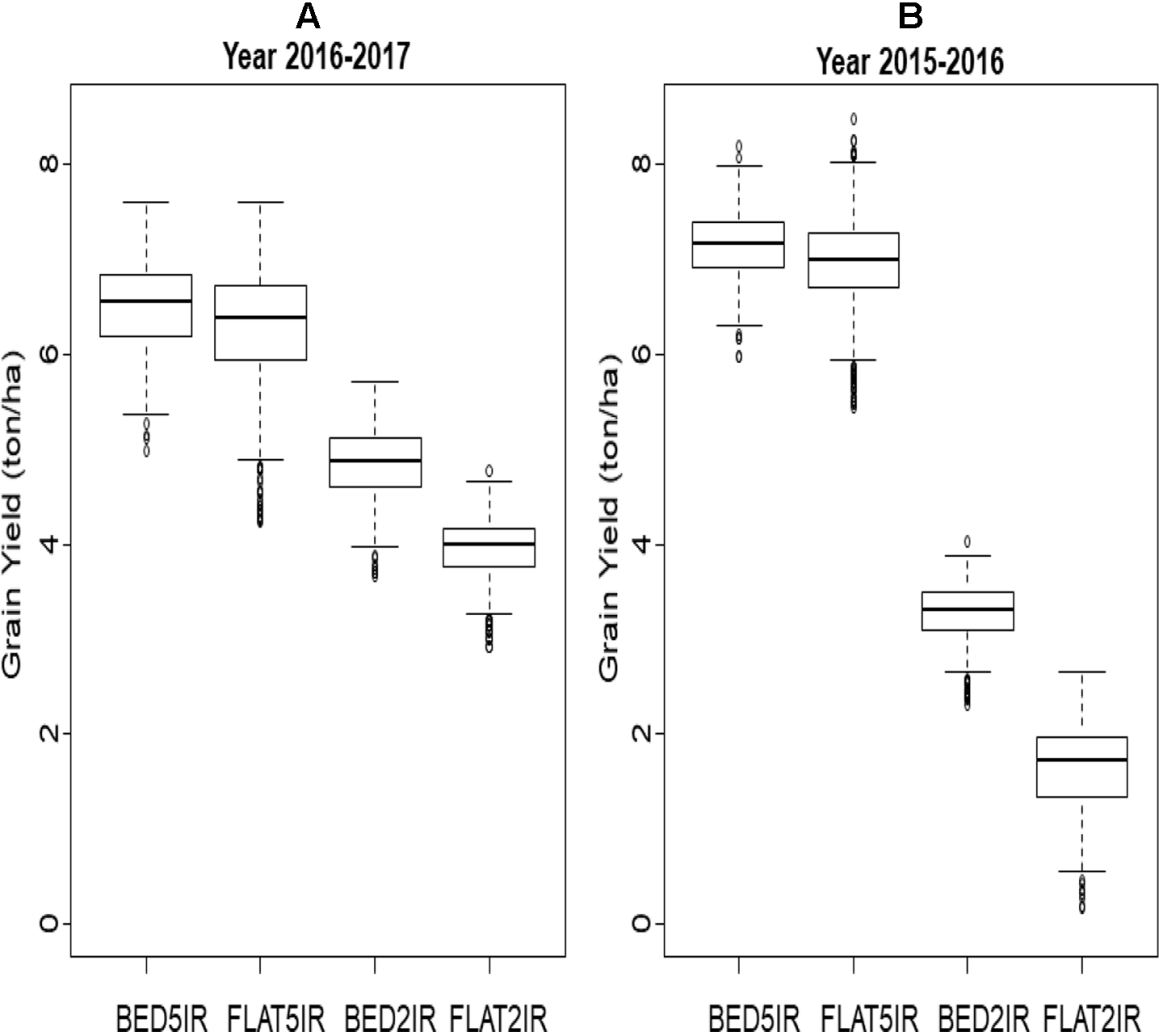
Box plot of grain yield (ton/ha) for four environments (BED5IR, FLAT5IR, BED2IR, and FLAT2IR) for **(A)** year 2016–2017 and **(B)** year 2015–2016.

A box plot of the grain yield of the four environments in each of the years (2015–2016 and 2016–2017) is displayed in **Figures 1A, B**. The two irrigated environments (BED5IR and FLAT5IR) in year 2015–2016 had similar productivity as in year 2016–2017, but the two drought environments (BED2IR and FLAT2IR) produced less grain yield in year 2015–2016 than in year 2016–2017, reflecting the year effect in the drought environments. The narrow-sense heritabilities based on the full model in Eq. (2) for grain yield of environments in year 2015–2016 were BED5IR=0.595, FLAT5IR=0.446, BED2IR=0.590, and FLAT2IR=0.744, and for environments in year 2016–2017 the narrow-sense heritability were BED5IR=0.547, FLAT5IR=0.603, BED2IR=0.565, and FLAT2IR=0.500.

In general, the phenotypic correlations between the four environments in each year were low except for the two drought environments BED2IR and FLAT2IR (0.609 in year 2015–2016 and 0.585 in year 2016–2017) (**Table 1**). The phenotypes of environment FLAT5IR were correlated with those obtained in environments BED2IR and FLAT2IR in year 2016–2017 at ∼0.44. The narrow-sense heritability of grain yield in all environment and year combinations was relatively high. Note that these heritability estimates were obtained using genomic markers for the single-environment and the multienvironment models. The heritability of grain yield for years 2016–2017 and 2015–2016 across all four environments were 0.72 and 0.82, respectively. The heritability for year 2016–2017 for the four environments ranged from 0.50 (FLAT2IR) to 0.60 (FLAT5IR), whereas for year 2015–2016, the heritability was 0.45 for FLAT5IR and 0.59 for BED5IR.

**Table 1:** Phenotypic correlations among four environments (BED5IR, BED2IR, FLAT5IR, and FLAT2IR) based on grain yield for year 2016–2017 (upper triangle) and year 2015–2016 (lower triangle).

Lower triangle\ upper triangle	BED5IR	FLAT5IR	BED2IR	FLAT2IR
BED5IR	1.000	0.098	0.131	0.006
FLAT5IR	0.260	1.000	0.443	0.446
BED2IR	0.214	0.093	1.000	0.585
FLAT2IR	0.094	0.113	0.609	1.000

### Genome-Based Prediction of the Single-Environment and Multienvironment Models

**Figure 2:**
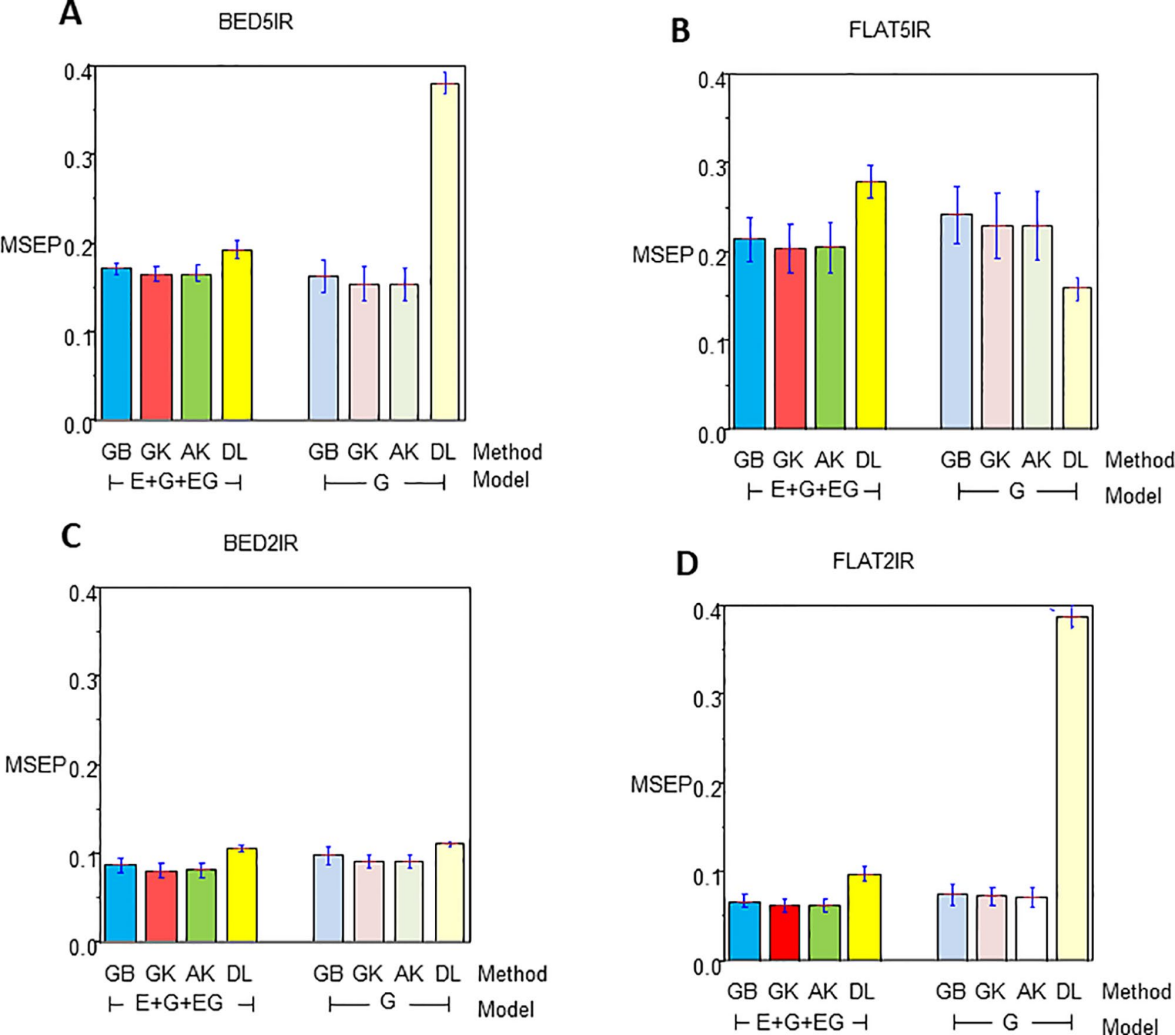
Mean squared error of the prediction for year 2016–2017 for single environment (G) and multienvironment (E+G+GE) models with kernels GB, GK, and AK and the deep learning (DL) method for environments **(A)** BED5IR, **(B)** FLAT5IR, **(C)** BED2IR, and **(D)** FLAT2IR.

**Figure 3:**
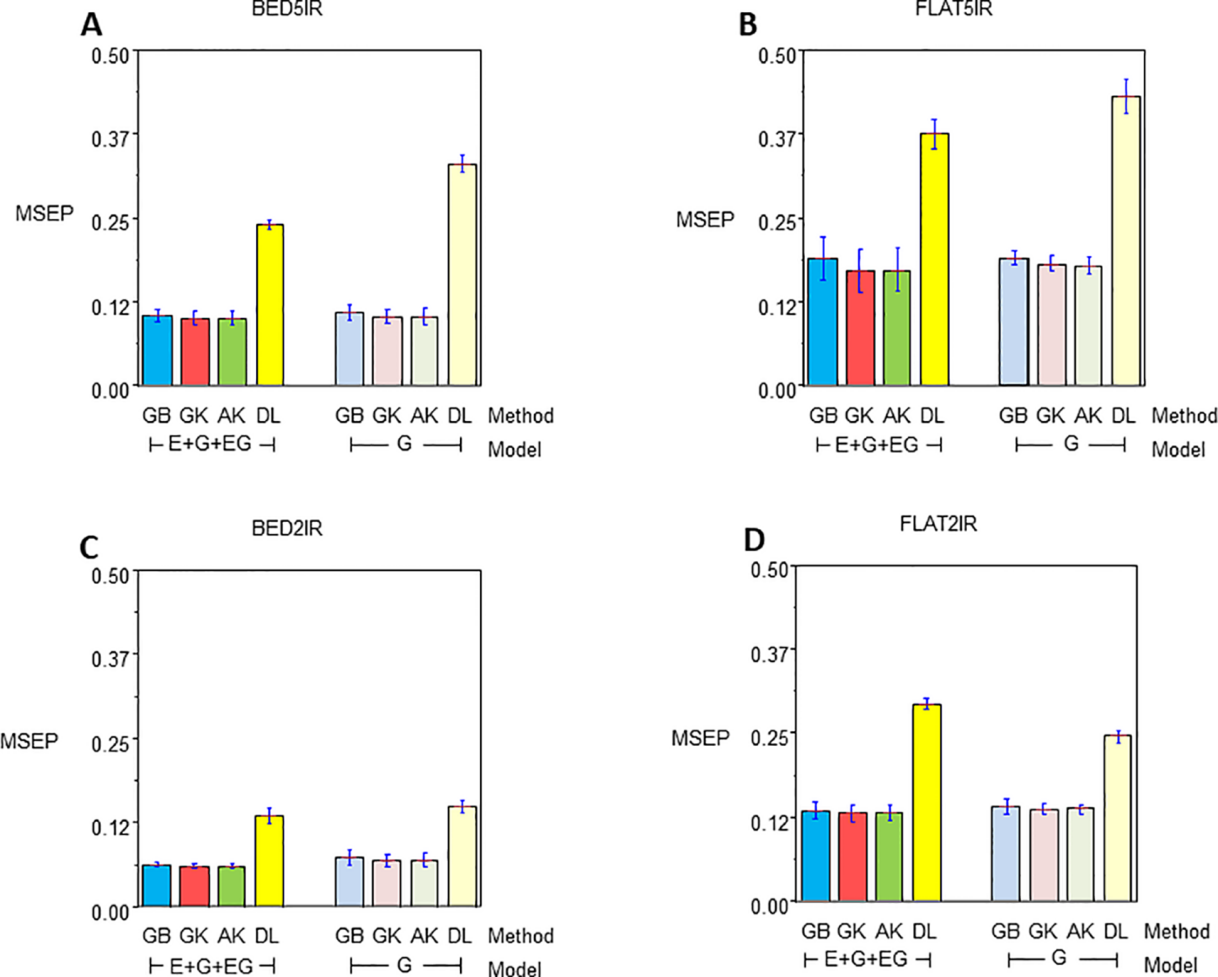
Mean squared error of the Prediction for year 2015–2016 for single environment (G) and multienvironment (E+G+GE) models with kernels GB, GK, and AK and the deep learning (DL) method for environments **(A)** BED5IR, **(B)** FLAT5IR, **(C)** BED2IR, and **(D)** FLAT2IR.

The results for year 2016–2017 for single-environment and multienvironment accuracies are shown in **Table 2** and **Figure 2**, whereas results for year 2015–2016 for single-environment and multienvironment accuracies are shown in **Table 3** and **Figure 3**.

**Table 2:** Average mean-squared-error prediction (MSEP) for year 2016–2017 of single environment (G) and multienvironment G×E models (E+G+GE) for predicting each environment comprising a combination of irrigation level (five irrigation, 5IR; two irrigations, 2IR) under two planting systems (FLAT and BED) for methods GBLUP (GB), Gaussian kernel (GK), arc-cosine (AK), (*l* is the number of layers of the deep kernel), and deep learning (DL).

		GB	GK	AK		DL
Model	Environment	MSEP	MSEP	MSEP	*l*	MSEP
E+G+EG	BED5IR	0.1719 (0.006)	**0.1656** (0.009)	0.1659 (0.009)	1	0.1924 (0.010)
E+G+EG	FLAT5IR	0.2144 (0.025)	**0.2040** (0.028)	0.2048 (0.028)	1	0.2797 (0.018)
E+G+EG	BED2IR	0.0867 (0.009)	**0.0807** (0.008)	0.0811 (0.008)	1	0.1066 (0.004)
E+G+EG	FLAT2IR	0.0669 (0.007)	**0.0624** (0.007)	0.0625 (0.007)	1	0.0977 (0.007)

G	BED5IR	0.1627 (0.019)	0.1545 (0.019)	**0.1544** (0.019)	5	0.3806 (0.012)
G	FLAT5IR	0.2415 (0.033)	0.2297 (0.037)	0.2297 (0.038)	4	**0.1589** (0.013)
G	BED2IR	0.0977 (0.010)	**0.0914** (0.008)	**0.0914** (0.008)	5	0.1110 (0.003)
G	FLAT2IR	0.0749 (0.012)	0.0723 (0.011)	**0.0718** (0.011)	5	0.3883 (0.012)
	Average	0.1396	**0.1326**	0.1327	–	0.2144

**Table 3:** Average mean-squared-error prediction (MSEP) for year 2015–2016 of single environment (G) and multienvironment G×E models (E+G+GE) for predicting each environment comprising a combination of irrigation level (five irrigation, 5IR; two irrigations, 2IR) under two planting system (FLAT and BED) for methods GBLUP (GB), Gaussian kernel (GK), arc-cosine (AK), (*l* is the number of layers of the deep kernel), and deep learning (DL).

		GB	GK	AK		DL
Model	Environment	MSEP	MSEP	MSEP	*l*	MSEP
E+G+EG	BED5IR	0.1048 (0.009)	**0.1007** (0.010)	**0.1007** (0.010)	1	0.2403 (0.007)
E+G+EG	FLAT5IR	0.1898 (0.032)	**0.1719** (0.032)	0.1729 (0.032)	1	0.3749 (0.023)
E+G+EG	BED2IR	0.0632 (0.004)	**0.0601** (0.004)	**0.0601** (0.004)	1	0.1355 (0.011)
E+G+EG	FLAT2IR	0.1349 (0.012)	**0.1318** (0.012)	0.1321 (0.012)	1	0.2931 (0.009)

G	BED5IR	0.1095 (0.011)	**0.1031** (0.011)	0.1036 (0.012)	5	0.3307 (0.0124)
G	FLAT5IR	0.1901 (0.010)	0.1819 (0.012)	**0.1792** (0.013)	4	0.4316 (0.025)
G	BED2IR	0.0729 (0.011)	**0.0690** (0.010)	0.0693 (0.010)	5	0.1495 (0.008)
G	FLAT2IR	0.1415 (0.012)	**0.1369** (0.008)	0.1377 (0.007)	5	0.2452 (0.009)
	Average	0.1288	**0.1194**	0.1195	–	0.2751

#### Year 2016–2017 Single-Environment Accuracy

The range of MSEP for the single-environment model (G) was between 0.0718 (AK for FLAT2IR) and 0.3883 (DL for FLAT2IR) (**Table 2** and **Figure 2**). Of the four methods implemented (GB, GK, AK, and DL), and the four environments, we found that the lowest MSEPs were obtained with the AK method in three environments, BED5IR, BED2IR, and FLAT2IR and the worst predictions were obtained with DL (except for FLAT5IR, where the best model was DL). The second best model was GK, which performed very similarly to AK (**Table 2**) for all the environments. Environments FLAT5IR and BED2IR had the same MSEP for both GK and AK (0.2297 and 0.0914, respectively).

The average MSEP for method GB was higher than for methods GK and AK, and the average MSEP of DL was also higher than that of any of the other three methods for all environments, except for environment FLAT5IR, where DL had the best prediction accuracy with an MSEP of 0.1589 (**Table 2** and **Figure 2B**). In addition, it is clear from **Figure 2C** that for environment BED2IR, the four methods had very similar prediction accuracies for the single-environment model (G) (GB=0.0977, GK=0.0914 AK=0.0914, and DL=0.1110).

#### Year 2016–2017 Multienvironment Accuracy

The best method in terms of MSEP was GK for all the environments under the G×E genomic model, while the lowest MSEP of 0.0624 was for environment FLAT2IR. The environment with the highest average MSEP was FLAT5IR for the DL method (0.2797) (**Table 2** and **Figure 2**). The AK kernel closely followed GK in terms of MSEP accuracy, ranging from 0.0625 (FLAT2IR) to 0.2048 (FLAT5IR). Methods GB and DL were the worst in terms of MSEP accuracy. Interestingly, except for GB, GK, and AK for environment BED5IR, and DL for environment FLAT5IR, the MSEP for model E+G+GE were smaller than the MSEP for model G for all four methods. The models including G×E are more precise than those including only the genomic effect (G), regardless of the method used. The differences between MSEP of method DL versus the MSEP of the other methods were much less for the E+G+GE model than those found for the single-environment model and especially for environments BED5IR and FLAT2IR, where the DL methods had high values for MSEP (see **Figures 2A, D**).

#### Year 2015–2016 Single-Environment Accuracy

Genome-enabled predictive abilities for the single-environment and multienvironment G×E models are given in **Table 3** and **Figure 3**. For the single-environment models (G), GK had the lowest MSEP in three environments (0.1031 for BED5IR, 0.0690 for BED2IR, and 0.1369 for FLAT2IR) but not for FLAT5IR, where AK was best (**Figure 3B**). The prediction accuracy of the linear kernel GB was lower than that of the nonlinear kernels (GK and AK), ranging from 0.0729 in BED2IR to 0.1901 in FLAT5IR. The DL accuracies of genome-based prediction were the worst, ranging from 0.1495 in BED2IR to 0.4316 in FLAT5IR.

**Figure 3** illustrates that the prediction accuracy of DL was not competitive with that of the other methods, which showed a very similar MSEP. The values of MSEP in environment BED2IR were the lowest across all the environments. The highest MSEP values were found in environment FLAT5IR.

#### Year 2015–2016 Multienvironment Accuracy

The best model in terms of MSEP was GK in all the environments under the G×E genomic model, with the lowest MSEP of 0.0601 in environment BED2IR. The environment with the highest average MSEP was FLAT5IR for the DL method (0.3749) (**Figure 3B**). AK had, together with GK, the two best prediction accuracies, in BED5IR (0.1007) and in BED2IR (0.0601) (**Table 3**). As already mentioned, kernel GK was also the best in FLAT5IR and in FLAT2IR (0.1318). Similar to previous cases, methods GB and DL were the worst in terms of MSEP accuracy. Results show that in all four environments except for FLAT2IR and DL, the MSEP for model E+G+GE were smaller than the MSEP for model G, for all four methods. The models including G×E were more precise than those that only included the genomic effect G.

Furthermore, in general, genome-based accuracy for year 2016–2017 was lower than genomic accuracy computed in year 2015–2016 (**Figure 2** vs. **Figure 3**). The DL method seemed to have more difficulties for learning from the data of year 2015–2016 than from the data of year 2016–2017. This may be partially due to the year effect and to the difficulty of optimizing the hyperparameters of the DL method in this year.

## Discussion

The two data sets included in this study represent two years of data with different wheat lines included in each year, but evaluated under the same experimental environments. Results show that the prediction accuracy of the same models, for instance DL, were very different across years. This may be a result of the different lines used in the two data sets, but more likely the year effects and differences in the G×E interaction. Using the average performance of the lines in each year and performing a two-year analysis may confound the year effect with the different line effects in each year. In order to avoid this possible confounding effect, we performed genomic G×E analyses across environments within each year.

### DL Method

DL is a branch of machine learning inspired by the functioning of the human brain. It is helping to automate many tasks that until some time ago only humans were able to perform (e.g., artificial intelligence and autonomous driving). Applications of DL are found in many domains, from social sciences to natural sciences. It is used for classifying exoplanets in astrophysics, for selecting human resources in enterprises, for detecting frauds in banks, and for detecting and classifying many types of cancers, among other things (Chollet and Allaire, 2017). In plant breeding, DL has been used to predict phenotypes of hybrids or lines for which only genomic information is available (Montesinos-López et al., 2018a; Montsinos-López, 2018b; Montsinos-López, 2019a; Montsinos-López, 2019b). However, the training process of DL models is challenging because successful implementation requires large data sets and a tuning process of many hyperparameters (number of hidden layers, number of neurons in each layer, type of activation function, number of epochs, batch size, learning rate, optimizer, etc.). For this reason, when a data set is not large enough, DL training is cumbersome and difficult, because part of the training data must be used to select the optimal combination of hyperparameters.

DL algorithms are flexible and generic and have attracted the interest of researchers working on genome-based predictions. However, the predictive ability of DL versus GBLUP has not been very convincing and not well studied, as pointed out by a recent review by Pérez-Enciso and Zingaretti (2019). Those authors mentioned that initial shallow single-layer neural networks are very competitive with penalized linear methods. However, what has not been addressed are the main difficulties of DL methods when appropriately tuning the hyperparameters and finding an optimal combination of them in order to achieve good genomic-enabled prediction accuracy without overfitting the data. In this study, authors have dedicated important efforts to fitting DL to the two data sets; however, the tuning process has been very difficult and cumbersome, and the results were not completely satisfying. Especially for the data set of the wheat lines from 2015–2016, the prediction accuracy was much smaller for DL than for any of the other models. We can speculate that investing a significant amount of extra time would have led to another set of hyperparameters resulting in better prediction accuracy.

### Optimization of the DL Algorithm

The network implemented in this study has no cycles or loops but is a feedforward topology where information moves in only one direction (forward) from the input nodes (prediction variables), through the hidden nodes, and to the output nodes (target variables). As previously described (see the *Material and Methods* section), we performed, for each of the 50 random partitions of the data, an optimization process for selecting the hyperparameters consisting of a grid search method to select the “optimal” set of hyperparameters for that specific partition of the random cross-validation; therefore, it was not possible to give one unique final set of estimated hyperparameters for implementing the DL method. Furthermore, the genomic-enabled prediction accuracy of the DL method will change for every random partition of the data due to the different ranges of the estimated hyperparameters.

Therefore, since the tuning of the DL algorithm is complex and biased for the different range of values of hyperparameters obtained in each of the 50 random partitions, it is reasonable to say that the optimization process for selecting the hyperparameters is suboptimal. This is related to the fact that the optimization process does not guarantee finding a global minimum but may end at a local minimum. This circumstance makes it difficult to tune DL methods.

### Deep Kernel Method

Due to the abovementioned difficulties, deep kernel methods that imitate DL methods are an appealing alternative because deep kernels also capture nonlinearity and complex interactions but do not need a complex tuning process, as does conventional DL. The kernel function induces nonlinear mapping from inputs *x* to feature vectors **Ф**(*x*_i_) by using the kernel trick function: k(*x* *_i_*, *x* *_i_*)=**Ф**(*x*)·(*x_i_^’^*) that mimics a single hidden layer or ANN model. Therefore, the iterated mapping of the following equation:

(5) $$k^{\left(l\right)}(x_{i},x_{i^{\prime }})=\overset{l\;times}{\overset{︷}{\Phi (\Phi (\ldots \Phi (x_{i})}})\cdot \overset{l\;times}{\overset{︷}{\Phi (\Phi (\ldots \Phi (x_{i^{\prime }})))}}$$

emulates the computation of a DL model (ANN with more hidden layers) where “*·*” represents the inner product. However, this iterative mapping does not lead to interesting results in linear kernels [*k*(***x*** *_i_*,***x*** *_i’_*)= ***x*** *_i_* *·* ***x*** *_i’_*], homogeneous polynomial kernels [*k*(***x*** *_i_*,***x*** *_i’_*)= (***x*** *_i_* *·* ***x*** *_i’_*)*^d^*] and Gaussian kernels $\left[k\left(x_{i},x_{i^{\prime }}\right)=e^{-\lambda ∥x_{i}-x_{i´}∥2}\right]$ (Cho and Saul, 2009). Applying the exponential function twice leads to a kernel which is different from GK, but the qualitative behavior will not be changed (Cho and Saul, 2009). However, in the AK, the recursion $k^{\left(l\right)}(x_{i},x_{i^{\prime }})=\overset{l\text{ times}}{\overset{︷}{\Phi (\Phi (\ldots \Phi (x_{i})}}))\cdot \overset{l\text{ times}}{\overset{︷}{\Phi (\Phi (\ldots \Phi (\;x_{i^{\prime }}))}})$, also alters the kernel qualitatively and mimics an ANN with more than one hidden layer. The results we obtained with AK were similar to those obtained with GK, but with the main advantage that a complex tuning process for choosing the bandwidth parameter across a grid is not required. We also found that GK and AK outperformed the DL method, which might be due to the fact that our data sets are not large enough for successful training of DL and that the main interaction structures within the data were known (G×E) and thus modeled directly.

It is important to point out that the AK deep kernel method is not completely exempt from a tuning process, since one needs to define the depth of the kernel (equivalent to the number of hidden layers). However, choosing such values is straightforward, since we only need to choose integers 1, 2, 3, 4, 5, etc. (Cho and Saul, 2009). We used the maximum marginal likelihood proposed by Cuevas et al. (2019) to select this parameter. As has been the case in many other studies, our results are not definitive, since we only compared the methods with two real data sets. For this reason, we encourage other scientists to do this benchmarking process with other types of data in order to increase the evidence of the prediction performance of these methods. Although our results are not conclusive, there is evidence that the AK (deep kernel) method competes well with DL and the GK, but with the main advantage that the tuning process is considerably less costly. For example, for cycle 2016–2017 with a marker matrix of 1040×8311, the average time for computing the squared distance for the basic GK was 105 s, whereas the computing time (using the same server) for the basic deep kernel AK1 (one layer) was 7 s. Similarly, the average computing time for selecting the bandwidth *h* for GK was, for each partition, 80 s. In contrast, the average time for selecting the number of layers for AK was 10 s. These differences increase (or decrease) exponentially as the size of the matrices to be used increases (or decreases). This advantage means that the AK method can be implemented in many statistical or machine learning software even by users with no background in statistics, computer science, or machine learning. The deep kernel method can be implemented and used more easily than DL models.

### On the Marginal Likelihood and the Number of Hidden Layers (Or Levels) of the AK Deep Kernel Method

To illustrate how the marginal likelihood changed with the number of hidden layers used in the AK deep kernel, we give the example of the marginal likelihood of the observations for environment BED5IR for year 2016–2017 for layers (*l*) 1 to 8. The corresponding values were -2109.017, -2104.825, -2102.632, -2101.585, -2101.228, -2101.305, -2101.669, and -2102.232, respectively. The maximum likelihood is reached at *l*=5 (-2101.228). Note that for method GB, the marginal likelihood is -2116.175, which is even lower than the first level (*l*=1) of the AK deep kernel (-2109.017).

## Conclusions

We performed a benchmarking study comparing a DL model with the AK deep kernel method, with the conventional GBLUP and with the nonlinear GK. We found that AK and GK performed very similar, but when taking the G×E interaction into account, GK constantly predicted best across all four environments and with both data sets. In general, AK and GK were better than GBLUP and DL. Our findings suggest that AK is an attractive alternative to DL and GK, since it offers competitive predictions at low costs in the tuning process. AK is a computationally simple model that makes it possible to emulate the behavior of DL networks with a large number of neurons. In general, the results of this study with respect to DL are not conclusive because the low performance of DL for year 2015–2016 may be partially a result of suboptimal hyperparameters.

## Data Availability Statement

All datasets generated for this study are included in the article/supplementary material.

## Author Contributions

All authors listed have made substantial, direct, and intellectual contribution to the work and approved it for publication.

## Funding

We are grateful for the financial support provided by the Bill & Melinda Gates Foundation and CIMMYT’s CGIAR CRP (maize and wheat), as well as the USAID projects (Cornell University and Kansas State University) that generated the CIMMYT wheat data analyzed in this study. We acknowledge the financial support provided by the Foundation for Research Levy on Agricultural Products (FFL) and the Agricultural Agreement Research Fund (JA) in Norway through NFR grant 267806.

## Conflict of Interest

The authors declare that the research was conducted in the absence of any commercial or financial relationships that could be construed as a potential conflict of interest.

## Appendix

### Basic Codes for AK

######## Equation (3)
### AK1.fun:Build the base kernel (AK1) of AK with level one
AK1.fun<-function(X){
     n<-nrow(X)
     cosalfa<-cor(t(X))
     angulo<-acos(cosalfa)
     mag<-sqrt(apply(X,1,function(x) crossprod(x)))
     sxy<-tcrossprod(mag)
     AK1<-(1/pi)*sxy*(sin(angulo)+(pi*matrix(1,n,n)-angulo)*cosalfa)
     AK1<-AK1/median(AK1)
     colnames(AK1)<-rownames(X)
     rownames(AK1)<-rownames(X)
     return(AK1)
      }  
#######  ### marg.AK function: Select the optimal recursion level 
marg.AK <- function(y,AK1,ml){
            lden.fun<-function(phi,nr,Uh,Sh,d){
            lden  <- -1/2*sum(log((1+phi*Sh)))-(nr-1)/2*log(sum(d^2/((1+phi*Sh))))
            lden <- -(lden)
            return(lden)
            }
           vero<-function(y,GC) {          
           Kh <- GC
           eigenKh <- eigen(Kh)
           nr<- length(which(eigenKh$val>1e-10))
           Uh <- eigenKh$vec[,1:nr]
           Sh <- eigenKh$val[1:nr]
           d <- t(Uh)%*%scale(y,scale=F)
           sol <-optimize(lden.fun,nr=nr,Uh=Uh,Sh=Sh,d=d,lower=c(0.0005),upper=c(200))
           phi<-sol[[1]]
           log.vero<--1/2*sum(log((1+phi*Sh)))-(nr-1)/2*log(sum(d^2/((1+phi*Sh))))
           return(log.vero)
           }
           GC<-AK1
           l<-1
           GC2<-GC
           vero1<-vero(y=y,GC=GC2)
           m<-0
           while( m==0 && (l<ml)){
                l<-l+1
                GC<-AK.fun(AK1=GC2,nl=1)
                GC2<-GC
                vero2<-vero(y=y,GC=GC2)
                if(vero2<vero1) m=1
                vero1<-vero2
                }
          return(l-1)
          }
######### Equation (4)
### Kernel.function: Build the AK kernel, with the base kernel (AK1) and the recursion level (nl)
AK.fun<-function(AK1,nl){
    n<-nrow(AK1)
    AK<-AK1
       for ( l in 1:nl){
           Aux<-tcrossprod(diag(AK))
           cosalfa<-AK*(Aux^(-1/2))
           cosa<-as.vector(cosalfa)
           cosa[which(cosalfa>1)]<-1
           angulo<-acos(cosa)
           angulo<-matrix(angulo,n,n)
           AK<-(1/pi)*(Aux^(1/2))*(sin(angulo)+(pi*matrix(1,n,n)-angulo)*cos(angulo))
       }
    AK<-AK/median(AK)
    rownames(AK)<-rownames(AK1)
    colnames(AK)<-colnames(AK1)
    return(AK)
  }
################################ Fitting the G (single enviroment) model
### Inputs: Matrix markers (X), observations  (y)
library (BGGE)
AK1<-AK1.fun(X)
trn<-!is.na(yna)
tst<-is.na(yna)
AKtrn<-AK1[trn,trn]
l<-marg.AK(y=y[trn],AK1=AKtrn,ml=30)
AK<-AK.fun(AK1=AK1,nl=l)
K<-list(list(Kernel=AK,Type="D"))
fit<-BGGE(y=yna,K=K,ne=1,ite=12000,burn=2000,thin=2,verbose=T)
cor(fit$yHat[tst],y[tst],use="pairwise.complete.obs")

Basic codes for DL
####Input and response variable
   X_trn=
    X_tst=
    y_trn=
    y_tst=
    Units_O=400
    Epoch_O= 1000
    Drop_O=0.05

###########specification of the Deep neural network #################
    model_Sec<-keras_model_sequential() 
    model_Sec %>% 
      layer_dense(units =Units_O , activation ="relu", input_shape = c(dim(X_trn)[2])) %>% 
      layer_dropout(rate =Drop_O) %>% 
      layer_dense(units =Units_O , activation ="relu") %>% 
      layer_dropout(rate =Drop_O) %>% 
      layer_dense(units =Units_O , activation ="relu") %>% 
      layer_dropout(rate =Drop_O) %>% 
      layer_dense(units =Units_O , activation ="relu") %>% 
      layer_dropout(rate =Drop_O) %>% 
      layer_dense(units =1) 
    ###########Compiling the model #################
    model_Sec %>% compile(
      loss = "mean_squared_error",
      optimizer = optimizer_adam(),
      metrics = c("mean_squared_error"))
    ###########Fitting the model #################
    ModelFited <-model_Sec %>% fit(
      X_trn, y_trn,
      epochs=Epoch_O, batch_size =56, verbose=0)

####Prediction of testing set ##########################
    Yhat=model_Sec %>% predict(X_tst)
    y_p=Yhat
    y_p_tst=as.numeric(y_p)
